# Our Hospitals and the National Relief Fund

**Published:** 1916-08-12

**Authors:** Harry Johnson

**Affiliations:** House Governor and Secretary. Leicester Royal Infirmary


### OUR HOSPITALS AND THE NATIONAL RELIEF FUND.

### A PROVINCIAL HOSPITAL MANAGER'S POINTS.

*To the Editor of* The Hospital.

Sir,—I am much indebted to you for sending me an advance proof of your leading article to appear in The Hospital of August 12, and I should like to express appreciation of the magnificent service you are rendering to the voluntary hospitals of the country by bringing forward their claims for grants from the National Relief Fund. I will give careful consideration to the article and to the questions which it raises in the next few days; but I should like to say that, on cursory consideration, the voluntary hospitals of the provinces (I cannot speak for London) will scarcely be doing themselves service by basing their claims for recognition by the Fund on their waiting lists, for as you have pointed out, voluntary hospitals have not been doing their duty if, by placing beds at the disposal of the military authorities, for which they receive monetary grants, they have thereby increased the number of civil patients on their waiting lists.

I venture to suggest, therefore, that it *should* be very much more advantageous to submit that, thanks to the magnificent spirit of hospital medical, nursing and administrative staffs, working to the utmost capacity of their institutions, useful service to the nation has been performed by the voluntary hospitals in the treatment of thousands of wounded soldiers, without serious diminution of usefulness to civilian patients, or increase of the waiting lists.

(It should not be forgotten that each county hospital, if it has had increased work brought to it by reason of the war, has also had its work diminished by the numbers of men who have joined the various branches of the Service, and whose medical treatment has been thereby provided by the State.)

Continuing this line of approach to the National Fund from the provincial hospitals' point of view, I would also suggest:—

(1) That facts be submitted with reference to the number of patients treated for physical inabilities, in order to enable them to join the Services. The institutions which have done this work on systematic lines have certainly been performing work of the utmost national importance.

(2) That figures be presented showing the number of resident medical officers of institutions who have given up their hospital duties to join the Forces, and the subsequent difficulty which there has been to secure successors to maintain efficiently the increasing work of the voluntary hospitals.

(3) That similar figures be presented from the nurse-training schools, as to the difficulties which have had to be surmounted in order that the senior and fully trained portion of hospital staffs might be released for military work, whose places have had to be taken by second- and third-year nurses, who have in turn been succeeded by large numbers of probationers. This part of the service of the voluntary hospitals and the responsibilities devolving on matrons and hospital sisters in the supervision and training of probationers has not been adequately recognised.

(4) That it is only by overcoming the greatest possible difficulties, medical, nursing, and administrative, owing to war's demands, that the work of the voluntary hospitals has been carried on.

(5) That the constantly increasing cost of administration, consequent upon the enhanced prices directly attributable to the war, has been very considerable, probably not less than 40 per cent. on pre-war times.

(6) That after this terrible conflict is over a tremendous amount of additional work will, by reason of their very efficiency, come to the hospitals from men who have been invalided from the Services, or discharged with a monetary pension as unfit for further service. No provision for medical attention then exists for them. The Insurance Act does not provide it, and the aid of the voluntary hospitals will be sought. This will probably mean the creation of new departments, medical and surgical, orthopædic, electro-therapeutic, etc., and an extension of service which at present hospital managers can only dimly foresee.

(7) That facts be presented and points submitted to prove, as you so rightly contend, that the National Insurance Act would have failed long since but for the splendid system of medical relief of serious cases which has been afforded by the hospitals.

There are other points to which I might refer did time allow of further consideration, but as you ask for the co-operation of hospital managers in course of post, I thought I should like to let you have a few early observations from the point of view of a provincial hospital manager.—I am, yours faithfully,

Harry Johnson,
House Governor and Secretary.

Leicester Royal Infirmary,

August 8, 1916.

*** Our correspondent, in properly insisting upon the duty of every voluntary hospital to keep down the numbers on its waiting list by providing adequately for its civil patients, seems to forget the varying circumstances under which the work has to be done at voluntary hospitals, restrictions of site sometimes being a pressing factor in this connection at the present time.—Ed. The Hospital.

